# Extended-Interval Gentamicin Dosing for Pulmonic Tularemia

**DOI:** 10.1155/2019/9870510

**Published:** 2019-09-26

**Authors:** Tyson Dietrich, Katelynn Garcia, Joe Strain, John Ashurst

**Affiliations:** ^1^Kingman Regional Medical Center, Department of Pharmacy, 3269 Stockton Hill Road, Kingman, AZ 86409, USA; ^2^Regional Health Rapid City Hospital, Department of Pharmacy, 353 Fairmont Blvd, Rapid City, SD 57701, USA; ^3^Kingman Regional Medical Center, Department of Emergency Medicine, 3269 Stockton Hill Road, Kingman, AZ 86409, USA

## Abstract

*Francisella tularensis* is a Gram-negative coccobacillus that is rarely encountered in clinical practice. Patients can present with cutaneous, pulmonary, cardiac, mucous membrane, or gastrointestinal involvement. A clinician should have a heightened suspicion in endemic areas or when outbreaks appear. Diagnosis is achieved through serological testing or polymerase chain reaction assays. Although historically the treatment of choice was streptomycin, gentamicin is now preferred due to its availability and relatively safer side effect profile with extended-interval dosing. Limited published evidence exists on the effectiveness of extended-interval gentamicin for tularemia. This case series describes four patients with pulmonic tularemia successfully treated with extended-interval dosing of gentamicin without treatment failure or relapse.

## 1. Introduction

*Francisella tularensis* is a Gram-negative coccobacillus that was first described in ground squirrels in 1912 by McCoy and Chapin under the name *Bacterium tularense* [1]. The oldest cases of tularemia dates back to a disease found in lemmings by Ziegler in Norway and a hare disease in Japan as early as 1818 [1]. However, the first case of verified human tularemia wasn't until 1914 [2]. Currently, cases of human tularemia are sporadic in the United States and typically occur between May and September each year [3]. Between 2001 and 2010 a total of 1,208 cases were reported to the Centers for Disease Control (CDC) with an incidence of 0.041 cases per 100,000 people [3]. The authors report four cases of pulmonary tularemia that were successfully treated with extended-interval dosing of gentamicin.

## 2. Patient Case 1

An 82-year-old male presented with generalized weakness, chest pain, cough with occasional clear sputum, and a weight loss of 15 pounds over the last month. On examination, he was febrile, tachypneic, and hypoxic with coarse breath sounds. A chest radiograph revealed a dense mass-like consolidation in the right lower lobe, and he was started on vancomycin, azithromycin, and ceftazidime. The patient noted contact with a stray cat and also a deceased mouse in his yard one week prior to becoming ill. On the third day of admission, a computerized tomography (CT) scan was performed and revealed an 8 cm consolidated infiltrate of the right lower lobe with possible underlying mass, enlarged right hilar and subcarnial lymph nodes, infiltrates in the right lower lobe, and a 2.6 cm consolidated infiltrate of the left lung base. A lung biopsy was positive for *F. tularensis* with postobstructive pneumonia. Antimicrobial therapy was changed to high-dose gentamicin 5 mg/kg intravenously every 36 hours (adjusted for renal function), ciprofloxacin, and metronidazole. The metronidazole was discontinued 4 days later and gentamicin was prescribed for a total course of 10 days in addition to 2 weeks of ciprofloxacin.

## 3. Patient Case 2

A 55-year-old female complaining of fever, shortness of breath, diaphoresis, and dyspnea on exertion was admitted due to a chest radiograph revealing a nodular density in the left upper lobe suspicious for a left-sided perihilar lung nodule measuring 12 mm. A CT scan confirmed a 15 mm nodule in the left upper lobe. She had a history of smoking but no other known risk factors. A biopsy of the nodular lesion was positive for Gram-negative coccobacillus that was confirmed to be *F. tularensis.* During the first 2 days of hospitalization the patient had fevers greater than 39°C. The patient was initiated on high-dose gentamicin 5 mg/kg intravenously every 24 hours with a 10-day course planned. She reported significant improvement and was afebrile at discharge.

## 4. Patient Case 3

An 81-year-old male presented with weakness, shortness of breath, fever, and cough with sputum production. He denied any exposure to rabbits, rodents, or birds. A chest radiograph on admission showed multifocal pulmonary opacifications compatible with known masses that appeared unchanged. A CT scan of the chest revealed multiple large mass-like consolidations in both lungs, with the right middle lobe being the only one not involved. A biopsy of one of the lung masses was positive for *F. tularensis.* The patient was initiated on gentamicin 5 mg/kg intravenously every 24 hours and ceftriaxone 2 grams intravenously once daily, which was discontinued after three days. A total of ten days of gentamicin was given, and the patient was discharged home.

## 5. Patient Case 4

A 57-year-old male was admitted due to flulike symptoms with generalized ache and malaise and fever. A CT scan of the chest showed a left-sided lung lesion, and a sputum culture was positive for *F. tularensis.* He denied any contact with animals, but noted that the field where he was working had many dead rabbits and he had recently experienced numerous insect bites over the last month. The patient was started on intravenous gentamicin 5 mg/kg every 24 hours and ceftriaxone 1 gram daily. A total of 10 days of intravenous gentamicin and 2 weeks of oral ciprofloxacin 500 mg twice daily was recommended by infectious disease.

## 6. Discussion

*F. tularensis* is a Gram-negative coccobacillus with four identified subspecies. *F. tularensis* subspecies *tularensis* (type A) is seen almost exclusively in North America and has been shown to be the most virulent strain in humans and animals [4]. Within the type A subspecies molecular subtyping revealed further subclassification into two subpopulations (Type A1 and A2) based upon geographical regions [2]. Type A2 is typically found west of the 100^th^ meridian line and causes infections in younger patients as compared to Type A1 [2]. Recently two genotypes of the A1 isolate were discovered: A1a and A1b. Both subtypes are found predominantly in the eastern United States with type A1a being the more invasive and lethal of the two [2]. *F tularensis* subspecies *holarctica* (type B) is clinically less virulent than type A and is the predominant strain found in Europe and Japan [2]. *F tularensis* subspecies *mediaasiatica* is the main subspecies found in Russia and Asia, while *F tularensis* subspecies *novicida* has only caused a handful of cases worldwide [2].

The clinical manifestations of tularemia are directly related to the infecting strain of *F. tularensis* and the mode of transmission. Ulceroglandular tularemia is the most common form and accounts for up to 80% of all diagnosed cases worldwide [5]. Typically, this form will begin with a skin lesion that eventually leads to localized lymphadenopathy [5]. Oropharyngeal tularemia is contracted through ingestion of infected food or water and can present with a painful exudative pharyngitis [5]. Typhoidal tularemia is most often caused by *F. tularensis* type A and carries a high morbidity and mortality [5]. It can present with fever, prostration, vomiting, and diarrhea and can progress to renal failure, meningitis, pericarditis, and rhabdomyolysis [5]. Pulmonic tularemia can occur either through the inhalation of organisms or through the hematogenous spread from another site [5]. The chest radiograph in pulmonary tularemia may show an infiltrate, pleural effusion, or lymphadenopathy [5].

Diagnosis of tularemia is based upon a high degree of clinical suspicion coupled with either serological testing or polymerase chain reaction (PCR) assays due to the difficulty of growing the organism on standard culture mediums [2]. A fourfold increase in the antibody levels between acute and convalescent immunoglobulin G (IgG) titers, an acute microagglutination titer of 1 : 128, or an acute tube agglutination titer of 1 : 160 suggests active infection but most individuals do not develop antibodies until several weeks into the illness [2]. PCR, however, carries a sensitivity of 78% and a specificity of 96% in diagnosing tularemia [2].

The mainstay of treatment for tularemia is the aminoglycosides [1, 2, 5]. Streptomycin 1 gram intramuscularly twice a day was historically the standard of care due to the high cure rate with no relapses seen with this treatment regimen [2, 5]. According to the World Health Organization, however, gentamicin is now the preferred antibiotic for those with severe tularemia requiring hospitalization due to its bactericidal properties against *F. tularensis* and its widespread availability [6]. Recommended gentamicin dosing is 5 mg/kg/day divided into dosing every 8 to 12 hours with a desirable peak concentration ranging from 8 to 12 mcg/mL with treatment duration ranging between 7 and 10 days in order to minimize relapses [7].

Little published data exists regarding extended-interval dosing of gentamicin in the treatment of tularemia. Extended-interval dosing is possible because of the postantibiotic effect in which bacterial growth is inhibited even though the drug concentrations fall below the minimum inhibitory concentration. Benefits of extended-interval dosing include increasing the peak concentrations which in turn enhance the bactericidal activity and clinical outcomes, patient convenience through fewer infusions, and less frequent monitoring. Currently, only one other report notes extended gentamicin dosing for the treatment of tularemia [8]. In this report, the authors kept serum peak levels between 8 and 10 mcg/mL and trough levels around 0.5 to 1.5 mcg/mL [8]. Much like our cases, none of the patients reported by Hassoun et al. reported treatment failure or adverse effects [8].

## 7. Conclusion

Tularemia is a rare disease that has global implications. Physicians must have a heightened suspicion for the disease when faced with patients from either an endemic area or during an acute outbreak. While streptomycin is listed as the drug of choice for treatment, this report describes several cases of successful treatment utilizing extended-interval dosing of gentamicin.
